# Interactive effect of sleep duration and trouble sleeping on frailty in chronic kidney disease: findings from NHANES, 2005–2018

**DOI:** 10.1080/0886022X.2025.2471008

**Published:** 2025-02-27

**Authors:** Xi-Zhe Zhang, Jiong-Ao Xiang, Jun-Jie Xu, Wen-Feng Wang, Yao-Dong Li

**Affiliations:** a The Second Clinical Medical College, Guangzhou University of Chinese Medicine, Guangzhou, Guangdong Province, China; b Second Clinical College, Wuhan University, Wuhan, Hubei Province, China; c Department of Dialysis, Zhuhai Hospital of Guangdong Provincial Hospital of Chinese Medicine, Guangdong Province, China; d Medical Affairs Department, The Fourth People’s Hospital of Shunde, Foshan (Wu Zhong Pei Memory Hospital of Shunde, Foshan), Foshan, Guangdong, China

**Keywords:** Sleep duration, trouble sleeping, interactive effect, chronic kidney disease (CKD), frailty

## Abstract

**Background:**

Both sleep disorders and chronic kidney disease (CKD) are recognized as significant public health concerns. In the general population, sleep disorders have been shown to be associated with frailty in the elderly. This study aims to evaluate the association between sleep duration and trouble sleeping with frailty in CKD patients, as well as the potential interactive effect between these two factors.

**Methods:**

This cross-sectional study analyzed data from the National Health and Nutrition Examination Survey (NHANES) spanning 2005–2018. Sleep duration and trouble sleeping was self-reported. Frailty was assessed using a 49-item frailty index. The associations between sleep duration, trouble sleeping, and frailty were analyzed using weighted multivariate logistic regression and restricted cubic splines. Subgroup analysis was conducted to determine the consistency of the study’s conclusions across various subgroups.

**Results:**

A total of 5,211 adult CKD patients were included in this analysis. Regression analysis results indicated that short sleep duration (OR = 1.364, 95% CI: 1.152–1.616), long sleep duration (OR = 1.648, 95% CI: 1.259–2.157), and trouble sleeping (OR = 2.572, 95% CI: 2.102–3.147) were significantly associated with an increased risk of frailty in CKD patients, with an interaction between sleep duration and trouble sleeping. Subgroup analysis revealed that the effects of trouble sleeping and sleep duration on frailty symptoms in CKD patients exhibit significant variation across age groups (*p* < 0.05 for interaction), with no notable differences observed in other subgroups. RCS results demonstrated a U-shaped relationship between frailty and sleep duration, with the lowest risk of frailty at 7.12 h of sleep.

**Conclusion:**

Our findings indicated that both sleep duration and trouble sleeping were significantly associated with frailty in CKD patients, with a notable interaction between these two factors. Therefore, prevention and intervention strategies for frailty in CKD patients should address multiple aspects of sleep health.

## 1. Introduction

Chronic Kidney Disease (CKD) has emerged as an increasingly serious global public health issue. Approximately 10% of the global adult population is affected by this condition, with approximately 1.2 million deaths attributed to it annually [1]. It is projected that by 2040, CKD will become the fifth leading cause of death globally [2]. Frailty is defined as a multidimensional syndrome characterized by diminished reserves in energy, physical capacity, cognition, and overall health, increasing susceptibility to stressors. Frailty predisposes individuals to adverse outcomes such as falls, delirium, and disability [3–5]. Previous research indicated that frailty was prevalent among adults with CKD and was associated with heightened risks of cardiovascular events and mortality [6,7]. Therefore, from a public health perspective, understanding the risk factors for frailty among individuals with CKD is particularly critical for the prevention and intervention of CKD.

Sleep is a primary determinant of physical and mental health, with sleep duration and the presence of trouble sleeping being key metrics of sleep health [8]. Short and poor-quality sleep are unrecognized risk factors for CKD progression [9]. Sleep disturbances are significantly associated with all-cause mortality in patients with CKD [10]. Previous studies showed that both sleep duration and trouble sleeping were closely associated with various diseases such as hypertension, type 2 diabetes, depression, suggesting potential interactive effect between these two sleeping metrics on health outcomes [11–14]. Sleep duration and trouble sleeping independently contribute to frailty as significant risk factors; extremes in sleep (less than 5 h or more than 9 h) or trouble sleeping could increase the risk of frailty in older adults and exacerbate frailty symptoms [15,16]. Potential mechanisms linking short sleep or insomnia with frailty involve alterations in neuroendocrine regulation, including decreased testosterone levels, chronic inflammation, elevated oxidative stress, and imbalanced growth hormone secretion. Conversely, long sleep durations are associated with increased melatonin and cortisol levels and decreased body temperature, which may influence frailty through effects on the immune system [17,18]. While existing literature has explored the relationship between sleep problems and frailty, particularly in dialysis patients [19,20], a comprehensive understanding of how sleep duration and trouble sleeping interact to influence frailty in the broader CKD population remains limited. Therefore, we hypothesize that in CKD patients, trouble sleeping and sleep duration may be associated with frailty. Furthermore, the coexistence of both trouble sleeping and abnormal sleep duration may synergistically increase the likelihood of frailty compared to experiencing either sleep disorder alone. This study aims to examine the association between sleep duration, trouble sleeping, and frailty in CKD utilizing a national representative data from the National Health and Nutrition Examination Survey (NHANES).

## 2. Methods

### 2.1. Data

This study analyzed data from the National Health and Nutrition Examination Survey (NHANES) from 2005 to 2018. NHANES is a nationally representative cross-sectional survey of non-institutionalized U.S. citizens conducted using a complex, multistage probability sampling design [21]. Data from NHANES have been released biennially since 1999, with detailed information released at https://www.cdc.gov/nchs/nhanes/index.htm. Data collection for NHANES has been approved by the Institutional Review Board (IRB) of the National Center for Health Statistics (NCHS), ensuring written informed consent from all participants. Secondary analysis of NHANES data was determined by the Institutional Review Board (IRB) of the University of North Texas Health Science Center to be ‘not human subjects research’. This cross-sectional study followed the Strengthening the Reporting of Observational Studies in Epidemiology (STROBE) reporting guidelines [22].

### 2.2. Study subjects

**Figure 1.**
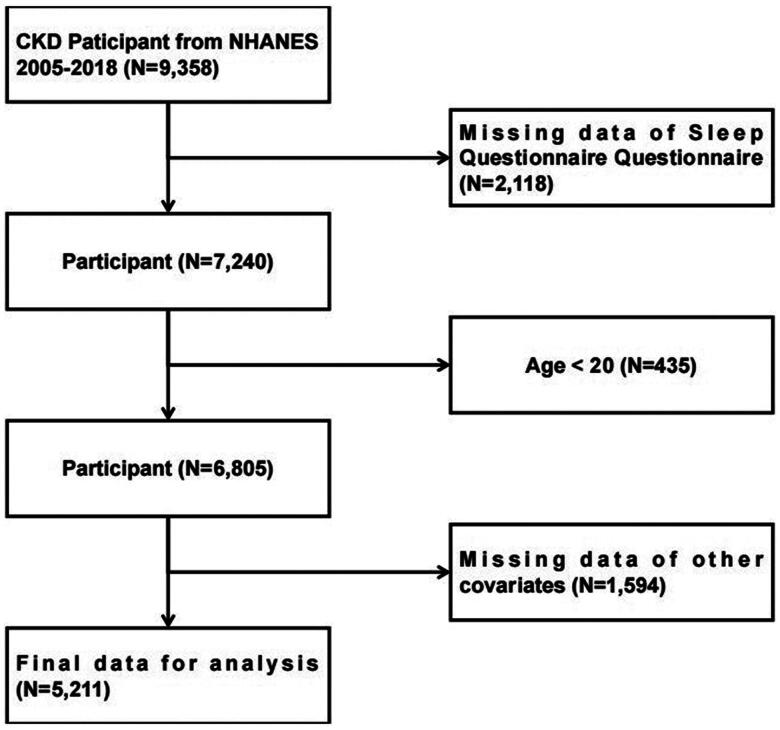
Flow chart of sample selection.

This study enrolled a total of 9358 subjects from the NHANES datasets spanning seven cycles from 2005 to 2018, who were diagnosed with CKD (defined as estimated glomerular filtration rate [eGFR] < 60 mL/min/1.73 m^2^and/or urinary albumin-to-creatinine ratio >30 mg/g) [23,24]. Exclusion criteria were: (1) missing sleep questionnaire data; (2) age <20 years; (3) missing data on marital status, poverty income ratio, education level, physical activity, smoking, alcohol consumption, body mass index, hypertension, diabetes, and other pertinent information; (4) >80% missing data on any frailty index. Ultimately, 5211 subject were selected for analysis (Figure 1).

### 2.3. Measures

#### 2.3.1. Measurement of sleep duration and trouble sleeping

Due to differences in sleep duration measurements between the periods 2005 and 2014 (5 cycles) and 2015 and 2018 (2 cycles), two procedures were employed to assess sleep duration. In the 2005–2014 cycles, sleep duration was recorded as hours ‘How many hours do you usually sleep on weekdays or workdays?’ For the 2015–2018 cycles, weekday sleep was assessed by inquiring about (1) bedtime and (2) wake-up time, while weekend or non-workday sleep was additionally recorded during the 2017–2018 period using (1) bedtime and (2) wake-up time questions. Sleep duration for the 2015–2016 period was calculated as the interval between bedtime and wake-up time. In the 2017–2018 period, assuming a typical weekly structure of 5 weekdays/workdays and 2 weekends/leisure days, weekday and weekend sleep times were estimated by weighted averaging [(weekday sleep duration *5 + weekend sleep duration * 2/7)], rounded to the nearest half-hour [25]. Although consensus on sleep duration definitions varies, epidemiological studies often define short sleep as ≤6 h and long sleep as ≥9 h [26,27].

Sleep difficulties were assessed via self-report by asking participants whether they had ever informed a doctor or other health professional about sleep problems (yes, no, refuse to answer, or don’t know); responses of refuse to answer or don’t know were recoded as missing values.

#### 2.3.2. Measurement of frailty

The degree of frailty was measured by the Frailty Index (FI), which was calculated according to the standardized procedure proposed by Samuel et al. [28]. We selected 49 relevant health indicators from NHANES to construct the FI, including cognitive, dependency, comorbidities, hospital utilization, medical conditions, and laboratory data (details in Supplementary Table 1). The FI scores, the number of deficits present divided by the total deficits considered, required completion of at least 80% of the indicators [28], yielding scores ranging from 0 to 1 where higher scores indicate greater frailty. Based on prior literature, FI ≤ 0.21 was categorized as non-frail and FI >0.21 as frail [29,30].

### 2.4. CKD stage

Serum creatinine was used to calculate eGFR with the Chronic Kidney Disease Epidemiology Collaboration (CKD-EPI) equation [31]. CKD was defined as estimated glomerular filtration rate [eGFR] < 60 mL/min/1.73 m^2^ and/or urinary albumin-to-creatinine ratio >30 mg/g) [23,24]. CKD is classified based on etiology, glomerular filtration rate (GFR) categories (G1–G5), and albuminuria categories (A1–A3), and is stratified into low risk, moderately increased risk, high risk, and very high risk [32].

### 2.5. Statistical analysis

All statistical analyses in this study were based on sample weights to generate prevalence estimates representative of the noninstitutionalized US population. Continuous variables were expressed as mean ± SD, whereas categorical variables were depicted as proportions. To compare inter-group differences, a weighted student’s *t*-test (for continuous variables) or a weighted chi-square test (for categorical variables) was performed.

Association between sleep duration and trouble sleeping with frailty was assessed using multivariable multinomial logistic regression analysis, evaluating odds ratios (OR) and 95% confidence intervals (CI). Covariates in this study included age, sex, race, marital status, education level, poverty income ratio (PIR), body mass index (BMI), smoking status, alcohol consumption, exercise habits, and comorbidities (e.g., hypertension, diabetes). Detailed information on covariates is provided in Supplementary Material 2(S2). Model 1 was unadjusted for covariates, using sleep duration and sleep difficulties as exposure factors and frailty symptoms as outcomes. Model 2 adjusted for age, sex, race, marital status, PIR, and education level. Model 3 further included smoking, alcohol consumption, vigorous and moderate exercise, BMI, diabetes, and hypertension as covariates based on Model 2. Model 4 extended Model 3 by incorporating interaction terms between sleep duration and trouble sleeping in the regression model. Nonlinear associations between sleep duration and frailty were analyzed using restricted cubic splines (RCS) with four knots set at the 5th, 35th, 65th, and 95th percentiles. Subgroup analyses were conducted to further explore whether there are differences in the effects of sleep duration and trouble sleeping on frailty symptoms among CKD patients with varying characteristics, such as age, sex, BMI, educational background, and comorbidities (including hyperlipidemia, hypertension, diabetes, cardiovascular disease (CVD), and osteoporosis). Additionally, ­sensitivity analyses were performed by adjusting for multiple variables, including CKD stage, serum phosphorus, serum calcium, serum potassium, triglycerides (TG), total cholesterol (TC), high-density lipoprotein cholesterol (HDL), osteoporosis, and hyperlipidemia, as well as excluding patients undergoing dialysis to further ensure the robustness of our findings.All statistical analyses were performed using R statistical software (version 4.1.0), and two-sided *p*-values <0.05 were considered statistically significant.

## 3. Results

### 3.1. Basic characteristics

This study selected 5,211 subjects from the NHANES database spanning 7 cycles between 2005 and 2018 (Table 1). Based on the frailty index employed in this study, the weighted sample represents a total U.S. population of 25,312,583 individuals, among whom 37% are classified as frail. Although the average sleep duration was similar between those with and without frailty symptoms, the weighted proportions of short sleep duration (32.5 vs. 26.1%) and long sleep duration (20.9 vs. 13.5%) were higher in the frailty group. The weighted proportion of trouble sleeping was also higher in the frailty group (47.1 vs. 24.2%) compared to those without frailty symptoms. Furthermore, compared to the non-frailty group, the frailty group had higher proportions of individuals with short sleep duration combined with trouble sleeping (19 vs. 7.6%) or long sleep duration combined with trouble sleeping (8.3 vs. 2.5%). Additionally, the frailty group had higher proportions of females, current or former smokers, individuals lacking exercise (including vigorous or moderate exercise), diabetes, and hypertension. Moreover, the frailty group had a higher average age (66 vs. 57.3 years), lower PIR (2.3 vs. 3.0), higher BMI (31.8 vs. 29.4), lower proportion of married or cohabiting individuals (52.5 vs. 62.8%), and lower education level (56% with high school education or below vs. 42.2%).

**Table 1.** Baseline characteristics of weighted sample by frail and non-frail groups.

	Total	No-frailty	Frailty	*p*-Value
*n* (sample size)	5,211	2,984	2,227	
*N* (weighted- sample size)	25,312,583	15,965,550	9,347,033	
Frailty score	0.195 ± 0.002	0.126 ± 0.001	0.312 ± 0.002	<0.0001
Sleep duration	7.273 ± 0.031	7.278 ± 0.036	7.265 ± 0.051	0.826
Sleep duration				<0.0001
>6, and <9	2,676 (55.285)	1,692 (60.402)	984 (46.545)	
< =6	1,682 (28.485)	885 (26.130)	797 (32.509)	
> =9	853 (16.230)	407 (13.469)	446 (20.946)	
Trouble sleeping				<0.0001
No	3,611 (67.362)	2,353 (75.832)	1,258 (52.894)	
Yes	1,600 (32.638)	631 (24.168)	969 (47.106)	
Trouble sleeping complaints & sleep duration interval				<0.0001
No trouble sleeping + sleep duration > 6h and <9h	1,986 (39.124)	1,366 (46.363)	620 (26.758)	
No trouble sleeping + sleep duration < = 6h	1,001 (16.671)	642 (18.523)	359 (13.508)	
No trouble sleeping + sleep duration > = 9h	624 (11.567)	345 (10.946)	279 (12.628)	
Trouble sleeping + sleep duration > 6h and <9h	690 (16.161)	326 (14.039)	364 (19.787)	
Trouble sleeping + sleep duration < = 6h	681 (11.814)	243 (7.607)	438 (19.000)	
Trouble sleeping + sleep duration > = 9h	229 (4.663)	62 (2.523)	167 (8.318)	
Age, year	60.550 ± 0.355	57.312 ± 0.460	66.080 ± 0.369	<0.0001
PIR	2.723 ± 0.043	2.982 ± 0.049	2.280 ± 0.051	<0.0001
BMI	30.307 ± 0.156	29.448 ± 0.193	31.775 ± 0.219	<0.0001
Sex				0.002
Male	2,520 (43.454)	1,496 (45.516)	1,024 (39.933)	
Female	2,691 (56.546)	1,488 (54.484)	1,203 (60.067)	
Race				<0.001
Mexican American	674 (6.758)	405 (6.980)	269 (6.380)	
Non-Hispanic Black	1,188 (12.088)	637 (10.474)	551 (14.844)	
Non-Hispanic White	2,550 (71.009)	1,448 (71.836)	1,102 (69.596)	
Other Hispanic	405 (4.342)	234 (4.646)	171 (3.823)	
Other race	394 (5.803)	260 (6.064)	134 (5.357)	
Marital status				<0.0001
Non-single	2,837 (58.976)	1,761 (62.788)	1,076 (52.465)	
Single	2,374 (41.024)	1,223 (37.212)	1,151 (47.535)	
Education				<0.0001
<High school	756 (8.530)	352 (6.328)	404 (12.291)	
High school	2,130 (38.888)	1,153 (35.849)	977 (44.080)	
>High school	2,325 (52.582)	1,479 (57.823)	846 (43.629)	
Smoke				<0.0001
Never	2,587 (50.728)	1,609 (54.981)	978 (43.463)	
Former	1,708 (32.763)	894 (30.035)	814 (37.422)	
Now	916 (16.509)	481 (14.983)	435 (19.115)	
Drinking				0.012
No	889 (14.208)	475 (12.950)	414 (16.356)	
Yes	4,322 (85.792)	2,509 (87.050)	1,813 (83.644)	
Vigorous physical activity				<0.0001
No	4,458 (83.096)	2,451 (79.513)	2,007 (89.216)	
Yes	753 (16.904)	533 (20.487)	220 (10.784)	
Moderate physical activity				<0.0001
No	3,534 (63.274)	1,892 (58.951)	1,642 (70.659)	
Yes	1,677 (36.726)	1,092 (41.049)	585 (29.341)	
Diabetes				<0.0001
No	3,089 (65.097)	2,131 (75.650)	958 (47.070)	
Yes	2,122 (34.903)	853 (24.350)	1,269 (52.930)	
Hypertension				<0.0001
** **No	1,454 (32.229)	1,152 (43.451)	302 (13.060)	
** **Yes	3,757 (67.771)	1,832 (56.549)	1,925 (86.940)	

For continuous variables: survey-weighted mean ± SE, *p*-value was by survey-weighted *t*-test.

For categorical variables, *n* (survey-weighted percentage) and *p*-value were measured using the survey-weighted Chi-square test.

PIR: poverty income ratio; BMI: body mass index.

### 3.2. Sleep duration and trouble sleeping associated with frailty

The association between sleep duration, trouble sleeping, and frailty symptoms is depicted in Table 2. Model 1 indicated that both short and long sleep durations, as well as trouble sleeping, were significantly associated with increased frailty risk. This association remained significant after further adjusting for multiple covariates in Models 2, 3, and 4.After conducting additional adjustments for CKD stage, serum phosphorus, serum calcium, serum potassium, TG, TC, HDL, osteoporosis, and hyperlipidemia, as well as excluding patients undergoing dialysis, the results remained significant (details in Supplementary Table 2).

**Table 2.** ORs for the associations of sleep duration and trouble sleeping with frailty symptoms.

	Model 1^a^ OR (95%CI) *p*-value	Model 2^b^ OR (95%CI) *p*-value	Model 3^c^ OR (95%CI) *p*-value	Model 4^d^ OR (95%CI) *p*-value
Sleep duration	0.995 (0.950, 1.041) 0.8259	0.954 (0.909, 1.001) 0.0602	0.966 (0.918, 1.016) 0.1818	1.006 (0.956, 1.058) 0.8170
Sleep duration				
>6, and <9	Ref.	Ref.	Ref.	Ref.
<= 6	1.615 (1.395, 1.868) <0.0001	1.633 (1.389, 1.920) <0.0001	1.538 (1.295, 1.826) <0.0001	1.364 (1.152, 1.616) 0.0005
>= 9	2.018 (1.609, 2.532) <0.0001	1.625 (1.308, 2.019) <0.0001	1.610 (1.244, 2.083) 0.0005	1.648 (1.259, 2.157) 0.0005
Trouble sleeping				
No	Ref.	Ref.	Ref.	Ref.
Yes	2.794 (2.352, 3.320) <0.0001	3.065 (2.545, 3.691) <0.0001	2.572 (2.102, 3.147) <0.0001	2.572 (2.102, 3.147) <0.0001

^a^Unadjusted model; ^b^adjusted for age, sex, race, marital status, PIR and educational level; ^c^adjusted for covariates in Model 2 and smoke, drinking, vigorous physical activity, moderate physical activity, BMI, diabetes, hypertension; ^d^in addition to all covariates included in model 3, sleep duration and trouble sleeping were each adjusted for the other.

**Figure 2.**
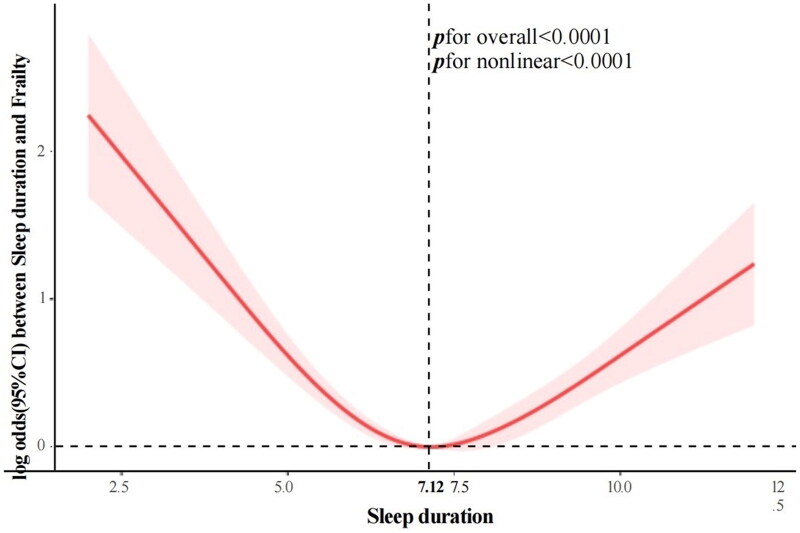
Log odds between sleep duration and frailty.

The nonlinear association between sleep duration and frailty was tested using restricted cubic splines (RCS), revealing a U-shaped relationship between the them (Figure 2). We validated this using segmented regression analysis (Table 3), showing that when sleep duration was less than 7.12 h, there was a negative correlation with frailty risk (Model 4: OR = 0.742, 95% CI: 0.676, 0.815, *p* < 0.0001). Conversely, when sleep duration exceeded 7.12 h, there was a positive correlation with frailty risk (Model 4: OR = 1.334, 95% CI: 1.176, 1.513, *p* < 0.0001).

**Table 3.** Nonlinear relationship between sleep duration and frailty.

	Sleep duration < =7.12	Sleep duration >7.12
	OR (95%CI) *p*-value	OR (95%CI) *p*-value
Model 1^a^	0.660 (0.607, 0.718) <0.0001	1.493 (1.327, 1.681) <0.0001
Model 2^b^	0.678 (0.618, 0.743) <0.0001	1.338 (1.194, 1.500) <0.0001
Model 3^c^	0.691 (0.629, 0.759) <0.0001	1.326 (1.170, 1.503) <0.0001
Model 4^d^	0.742 (0.676, 0.815) <0.0001	1.334 (1.176, 1.513) <0.0001

^a^Unadjusted model; ^b^adjusted for age, sex, race, marital status, PIR and educational level; ^c^adjusted for covariates in Model 2 and smoke, drinking, vigorous physical activity, moderate physical activity, BMI, diabetes, hypertension; ^d^in addition to all covariates included in model 3 and trouble sleeping.

### 3.3. Interactive effects of sleep duration and trouble sleeping

**Figure 3.**
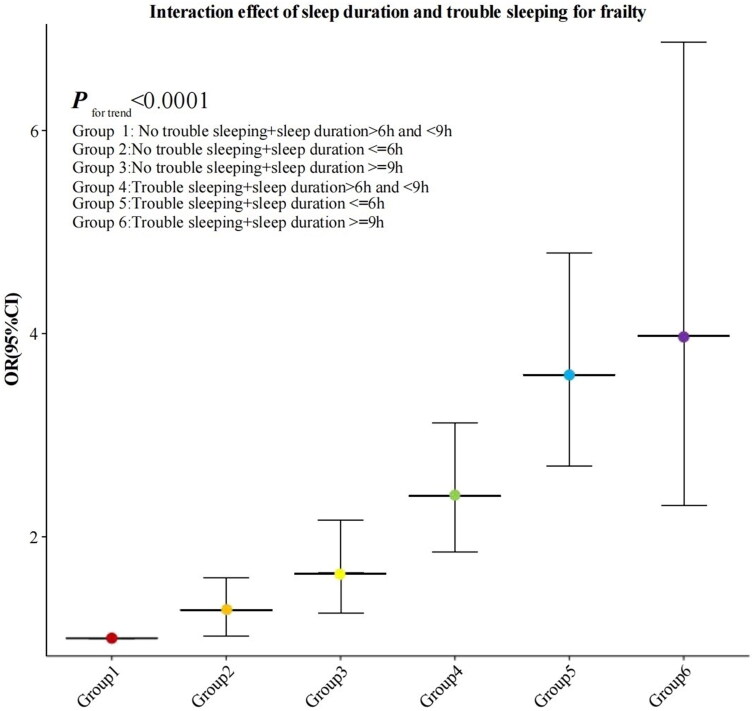
Forest plot of interaction effect of sleep duration and trouble sleeping for frailty.

The interactive effects of sleep duration and trouble sleeping on frailty symptoms are detailed in Table 4. Setting individuals with normal sleep duration and without trouble sleeping as the reference group, we observed that the combined groups of short or long sleep duration with trouble sleeping had a higher risk of frailty compared to those with only short or long sleep duration. In the fully adjusted regression model, the odds ratio (OR) for short sleep duration without trouble sleeping was 1.276 (95% CI: 1.021, 1.595), for long sleep duration without trouble sleeping was 1.639 (95% CI: 1.242, 2.162), for normal sleep duration with trouble sleeping was 2.402 (95% CI: 1.849, 3.120), for short sleep duration with trouble sleeping was 3.594 (95% CI: 2.695, 4.793), and for long sleep duration with trouble sleeping was 3.975 (95% CI: 2.301, 6.869), all statistically significant. After conducting additional adjustments for CKD stage, serum phosphorus, serum calcium, serum potassium, TG, TC, HDL, osteoporosis, and hyperlipidemia, as well as excluding patients undergoing dialysis, the results remained significant (details in Supplementary Table 3). The interaction effects of sleep duration and trouble sleeping are visually represented in a forest plot (Figure 3, *p* trend <0.0001).

**Table 4.** Interaction effect of sleep duration and trouble sleeping for frailty.

		Model 1^a^ OR (95%CI) *p*-value	Model 2^b^ OR (95%CI) *p*-value	Model 3^c^ OR (95%CI) *p*-value
Sleep duration	Trouble sleeping			
>6, and <9	No	Ref.	Ref.	Ref.
<= 6		1.264 (1.042, 1.533) 0.0194	1.316 (1.055, 1.643) 0.0170	1.276 (1.021, 1.595) 0.0351
>= 9		1.999 (1.562, 2.558) <0.0001	1.640 (1.294, 2.077) 0.0001	1.639 (1.242, 2.162) 0.0008
>6, and <9	Yes	2.442 (1.931, 3.088) <0.0001	2.805 (2.204, 3.569) <0.0001	2.402 (1.849, 3.120) <0.0001
<= 6		4.328 (3.414, 5.486) <0.0001	4.451 (3.410, 5.810) <0.0001	3.594 (2.695, 4.793) <0.0001
>= 9		5.713 (3.615, 9.029) <0.0001	4.975 (3.124, 7.922) <0.0001	3.975 (2.301, 6.869) <0.0001
*p* For trend		<0.0001	<0.0001	<0.0001

^a^Unadjusted model; ^b^adjusted for age, sex, race, marital status, PIR and educational level; ^c^adjusted for covariates in Model 2 and smoke, drinking, vigorous physical activity, moderate physical activity, BMI, diabetes, hypertension.

### 3.4. Subgroup analysis

Subgroup analyses and interaction tests were based on variables including age, sex, BMI, educational background, and comorbidities (including hyperlipidemia, hypertension, diabetes, CVD, and osteoporosis. This further confirms that the effects of trouble sleeping and sleep duration on the frailty symptoms of CKD patients exhibits significant differences across different age groups (*p* for interaction <0.05), while such differences are not significant among other subgroups (Table 5). Setting CKD patients with normal sleep duration and without trouble sleeping as the reference group, we observed that among patients under the age of 60, the odds ratio (OR) for short sleep duration without trouble sleeping was 1.944 (95% confidence interval CI: 1.223, 3.091), and for short sleep duration with trouble sleeping was 7.734 (95% CI: 4.802, 12.458).

**Table 5.** Subgroup analysis.

Character	No trouble sleeping + sleep duration > 6h and <9h	No trouble sleeping + sleep duration <= 6h	p	No trouble sleeping + sleep duration >= 9h	p	Trouble sleeping + sleep duration > 6h and <9h	*p*	Trouble sleeping + sleep duration <= 6h	p	Trouble sleeping + sleep duration >= 9h	p	*p* For trend	*p* For interaction
Age													0.004
<60	Ref	1.944 (1.223, 3.091)	0.005	1.626 (0.719, 3.676)	0.24	3.699 (2.265, 6.040)	<0.0001	7.734 (4.802, 12.458)	<0.0001	2.413 (0.741, 7.859)	0.142	<0.0001	
>= 60	Ref	1.067 (0.835, 1.363)	0.6	1.596 (1.223, 2.082)	<0.001	1.999 (1.412, 2.830)	<0.001	2.255 (1.693, 3.004)	<0.0001	4.797 (2.826, 8.142)	<0.0001	<0.0001	
Sex													0.708
Male	Ref	1.376 (1.000, 1.894)	0.05	1.932 (1.369, 2.727)	<0.001	2.611 (1.792, 3.805)	<0.0001	4.669 (3.140, 6.944)	<0.0001	4.477 (1.836, 10.918)	0.001	<0.0001	
Female	Ref	1.238 (0.887, 1.727)	0.206	1.420 (0.948, 2.128)	0.088	2.340 (1.565, 3.498)	<0.0001	3.089 (2.057, 4.640)	<0.0001	3.830 (1.905, 7.701)	<0.001	<0.0001	
BMI													0.677
<25	Ref	1.141 (0.754, 1.726)	0.528	1.379 (0.816, 2.328)	0.226	2.539 (1.574, 4.095)	<0.001	2.745 (1.370, 5.501)	0.005	3.890 (1.654, 9.153)	0.002	<0.0001	
>= 25, <30	Ref	1.444 (0.941, 2.215)	0.092	1.539 (1.015, 2.331)	0.042	2.192 (1.333, 3.603)	0.002	4.092 (2.610, 6.418)	<0.0001	8.970 (3.872, 20.784)	<0.0001	<0.0001	
>= 30	Ref	1.223 (0.890, 1.681)	0.211	1.825 (1.116, 2.985)	0.017	2.426 (1.645, 3.578)	<0.0001	3.851 (2.635, 5.627)	<0.0001	2.737 (1.398, 5.359)	0.004	<0.0001	
Education													0.464
<High school	Ref	1.211 (0.673, 2.181)	0.518	1.159 (0.551, 2.435)	0.693	4.093 (1.811, 9.253)	<0.001	2.841 (1.486, 5.430)	0.002	13.934 (3.097, 62.697)	<0.001	<0.0001	
High school	Ref	1.215 (0.845, 1.747)	0.289	1.436 (0.952, 2.165)	0.084	2.185 (1.453, 3.287)	<0.001	3.493 (2.192, 5.566)	<0.0001	2.926 (1.579, 5.422)	<0.001	<0.0001	
>High school	Ref	1.308 (0.872, 1.962)	0.191	2.147 (1.361, 3.388)	0.001	2.542 (1.685, 3.834)	<0.0001	3.992 (2.684, 5.937)	<0.0001	4.891 (1.838, 13.011)	0.002	<0.0001	
Hyperlipidemia													0.109
No	Ref	1.504 (0.811, 2.789)	0.193	1.364 (0.728, 2.559)	0.329	1.461 (0.835, 2.558)	0.181	5.672 (2.620, 12.280)	<0.0001	6.028 (1.871, 19.422)	0.003	<0.0001	
Yes	Ref	1.227 (0.961, 1.566)	0.1	1.645 (1.203, 2.250)	0.002	2.532 (1.866, 3.436)	<0.0001	3.334 (2.442, 4.552)	<0.0001	3.664 (2.053, 6.536)	<0.0001	<0.0001	
Hypertension													0.134
No	Ref	1.551 (0.974, 2.468)	0.064	1.134 (0.529, 2.432)	0.744	3.459 (1.979, 6.044)	<0.0001	5.050 (2.770, 9.206)	<0.0001	2.038 (0.739, 5.621)	0.167	<0.0001	
Yes	Ref	1.208 (0.923, 1.580)	0.167	1.804 (1.326, 2.454)	<0.001	2.213 (1.640, 2.985)	<0.0001	3.264 (2.401, 4.439)	<0.0001	4.609 (2.310, 9.196)	<0.0001	<0.0001	
Diabetes													0.093
No	Ref	1.249 (0.924, 1.688)	0.146	1.859 (1.283, 2.693)	0.001	2.800 (2.068, 3.791)	<0.0001	4.156 (2.930, 5.896)	<0.0001	6.474 (3.342, 12.539)	<0.0001	<0.0001	
Yes	Ref	1.320 (0.904, 1.928)	0.148	1.357 (0.894, 2.059)	0.15	1.943 (1.264, 2.988)	0.003	2.837 (1.834, 4.388)	<0.0001	2.239 (1.166, 4.298)	0.016	<0.0001	
CVD													0.881
No	Ref	1.341 (1.001, 1.794)	0.049	1.606 (1.118, 2.309)	0.011	2.349 (1.682, 3.283)	<0.0001	3.595 (2.521, 5.125)	<0.0001	3.727 (1.898, 7.318)	<0.001	<0.0001	
Yes	Ref	1.059 (0.669, 1.675)	0.805	1.599 (0.958, 2.671)	0.072	2.726 (1.624, 4.577)	<0.001	3.179 (1.860, 5.434)	<0.0001	4.465 (1.837, 10.851)	0.001	<0.0001	
Osteoporosis													0.498
Normal	Ref	1.550 (1.006, 2.389)	0.047	1.488 (0.846, 2.619)	0.164	2.738 (1.591, 4.713)	<0.001	4.151 (2.772, 6.218)	<0.0001	2.638 (0.735, 9.472)	0.134	<0.0001	
Osteopenia	Ref	1.241 (0.792, 1.944)	0.34	1.576 (0.894, 2.779)	0.114	1.843 (0.978, 3.473)	0.058	3.518 (2.170, 5.703)	<0.0001	5.321 (2.090, 13.550)	<0.001	<0.0001	
Osteoporosis	Ref	2.076 (0.715, 6.025)	0.171	3.217 (1.153, 8.974)	0.027	6.484 (1.923, 21.860)	0.004	3.826 (1.290, 11.347)	0.018	8.970 (0.930, 86.563)	0.057	0.002	

## 4. Discussion

Previous research on the relationship between sleep issues and frailty has predominantly focused on general elderly populations. This study represents the first attempt to utilize nationally representative sample data to investigate the association between sleep duration, trouble sleeping, and frailty symptoms among CKD patients. A total of 5,211 subjects were involved in this study. In all adjusted models, we found that both short (≤6 h) and long (≥9 h) sleep duration, as well as trouble sleeping, were significantly associated with increased frailty risk among CKD patients. Moreover, sleep duration and trouble sleeping demonstrated an interactive effect on frailty symptoms in this population. Our findings indicated that among CKD patients, short sleep duration alone increased the risk of frailty by 36%, while long sleep duration increased it by 65%. Having trouble sleeping raised frailty risk by 157%. We also observed a U-shaped relationship between sleep duration and frailty risk, with the lowest risk observed at 7.12 h of sleep; risks gradually increased with decreasing or increasing sleep durations. Additionally, CKD patients with normal sleep duration combined with trouble sleeping had 1.4 times the frailty risk compared to those without sleep difficulties, while combining short or long sleep duration with trouble sleeping resulted in over three times the frailty risk compared to having short or long sleep duration alone. Subgroup analysis reveals that while trouble sleeping and sleep duration similarly affect the symptoms of frailty in chronic kidney disease (CKD) patients, significant differences in these effects are observed across age groups (interaction *p* < 0.05). In contrast, no significant differences are noted among other subgroups. These findings underscored the synergistic interactions across different dimensions of sleep health and their close association with frailty in CKD patients.

While previous studies have varied in their definitions of sleep duration, most confirm a significant association between sleep issues and frailty in older adults [17]. A study on elderly Japanese individuals indicated that both short (≤6 h) and long sleep duration (≥9 h) were correlated with frailty [33]. A prospective cohort study of Chinese adults aged 80 years and older also found a U-shaped non-linear relationship between sleep duration and frailty risk, suggesting that the risk of frailty was relatively lower when sleep duration was between 6.5 and 8.5 h [34]. Our research revealed that trouble sleeping and sleep duration significantly impact frailty symptoms in CKD patients, with notable differences across various age groups. This finding could be associated with the varying incidence rates of CKD within different age populations [35]. A meta-analysis further demonstrated significant associations between both long and short sleep durations and increased frailty risk in both male and female populations [36]. However, literature on the relationship between sleep issues and frailty is not entirely consistent. For instance, a prospective cohort study involving 2505 men in the United States found no significant association between sleep duration and risk of frailty or mortality [37]. Another study indicated that insomnia, poor sleep quality, and sleep duration of less than 5 h increased the likelihood of frailty in women but not in men [38]. Additionally, research has shown that behaviors such as short sleep duration, insomnia, or snoring increase the risk of worsening frailty status, whereas no association was found with long sleep duration [17]. These inconsistencies among study findings may stem from differences in frailty measurement, assessment of sleep duration and its definitions, or baseline differences across different study cohorts. Our study results align with the majority of previous research, indicating not only a straightforward association between sleep duration and trouble sleeping with frailty in CKD patients but also highlighting complex interactions between sleep duration and trouble sleeping.

Previous studies have suggested that sleep disorders may increase the risk of frailty in CKD patients [39]. However, most of these studies have not concurrently examined sleep duration and trouble sleeping as coexisting risk factors for frailty. Consequently, the specific contributions and combined effects of these sleep parameters on frailty within the CKD population remain inadequately quantified. Unlike the general population, CKD patients, as a characteristic pathological group, typically exhibit lower sleep duration and poorer sleep quality, even in early CKD stages. Among patients with end-stage kidney disease (ESKD), the overall prevalence of various sleep disorders ranges from 45 to 80%, affecting half of early CKD patients [40]. Compared to non-CKD patients, CKD patients have a significantly higher likelihood of experiencing frailty [41]. As renal function declines, the risk of frailty further increases, and frailty is significantly associated with various stages of chronic kidney disease [41–44]. A meta-analysis has identified frailty as a critical predictor of mortality, hospitalization, and fall rates in CKD patients [45]. However, frailty is a dynamic process; individuals experiencing frailty may recover to a non-frail state, but without intervention, it may progress to long-term frailty [46]. Therefore, investigating whether sleep duration and trouble sleeping contribute to frailty in this specific population with CKD is of significant clinical importance.

The biological mechanisms linking sleep issues to frailty remain unclear. Firstly, dysregulation in neuroendocrine pathways may be a potential mechanism through which sleep problems contribute to frailty. Chronic inflammation, decreased testosterone levels, increased oxidative stress, and growth hormone imbalance may underlie the association between short sleep duration and frailty [17,47]. Conversely, excessive sleep duration can elevate melatonin and cortisol levels, decrease body temperature, disrupt the immune system, and also lead to frailty [48]. Secondly, both sleep problems and frailty are associated with elevated inflammatory markers such as CRP and interleukin-6 (IL-6) [49,50], suggesting inflammation may play a crucial role in the relationship between sleep and frailty. Additionally, some research indicated direct relationships between sleep parameters and adipokine concentrations [36]. Compared to non-frail individuals, those experiencing frailty exhibited significantly higher plasma levels of adiponectin [51,52]. Sleep issues may increase the risk of frailty by influencing adipokines. Notably, numerous studies highlighted the association between CKD and inflammation, with inflammation being a significant factor in disease progression and complications [1,53]. Compared to healthy individuals, CKD patients in stages 3–5 show increased oxidative stress and acute-phase inflammation, with significant differences in plasma inflammatory markers like CRP and IL-6 [54]. Sleep disorders may exacerbate inflammatory responses, further worsening frailty. Furthermore, CKD is also associated with elevated levels of adipose-derived molecules such as leptin, adiponectin, angiotensin II, IL-6, tumor necrosis factor (TNF), and metabolic disturbances [55]. The above evidence points to the intricate interplay among metabolic changes induced by CKD, sleep disorders, and frailty. Future research should delve deeper into the underlying biological mechanisms.

Frailty has been confirmed to be associated with negative outcomes in CKD such as cardiovascular disease, all-cause mortality, and all-cause hospitalization [45]. Therefore, gaining a deeper understanding of modifiable risk factors for frailty symptoms in CKD is crucial for clinical practice. This study substantiates the independent and interactive effects of sleep duration and trouble sleeping on frailty in CKD. It is noteworthy that ‘trouble sleeping’ or ‘sleep difficulty’ are broad medical terms that may encompass insomnia disorders, sleep environment issues, or other factors, and can manifest in various forms such as difficulty falling asleep and maintaining sleep [56]. Thus, caution should be exercised when comparing our findings with studies focused on insomnia. we also suggest that future studies should focus on the contemporaneous prevalence rates and therapeutic approaches to better reflect the current landscape of health and disease. Additionally, CKD patients often present with comorbidities, including hypertension, diabetes, and cardiovascular diseases. Comorbidities may confound the association between sleep and frailty [57], necessitating further exploration of these complex relationships. Furthermore, research indicates associations between either excessively long or short sleep durations, insomnia, or poor sleep quality and frailty in women [38,58]. Our study also found a higher proportion of females among frail CKD patients, suggesting avenues for further exploration into gender differences and potential underlying reasons.

The current findings hold significant implications for public health. We identified the differential impacts of various dimensions of sleep health on frailty in CKD patients, emphasizing the need for clinicians to routinely assess sleep health in CKD patients, actively screening for individuals with concurrent trouble sleeping and abnormal sleep durations, and providing preventive interventions tailored to this group. Strategies can also be devised to mitigate the simultaneous occurrence of trouble sleeping and extremes of sleep duration, thereby alleviating the harm caused by their interaction.

### 4.1. Advantages and limitations

This study has specific strengths. Although the literature on the relationship between sleep and frailty is expanding, especially among dialysis patients, our research offers a comprehensive and up-to-date analysis focusing on the specific impacts of sleep duration, trouble sleeping, and their interactive effects on the risk of frailty within the broader CKD population. We utilized a large sample from the nationally representative NHANES survey, adjusting for major potential confounders to assess their association with frailty in CKD patients. Our findings not only augment the existing body of knowledge but also fill a critical gap in understanding the intricate interplay between sleep health and frailty in CKD, a factor of growing relevance in contemporary clinical practice. However, our study also has several limitations. First, the cross-sectional study design limits our ability to draw causal inferences, thereby constraining the strength of our conclusions. Second, NHANES only provides a single measurement of serum creatinine and urine albumin, which may introduce bias in diagnosing CKD. Third, sleep duration, trouble sleeping, and other variables such as medical history and healthcare were self-reported, which may lead to biases or inaccuracies in certain estimates and potentially affect the reliability of the study’s findings. Fourth, key factors influencing frailty in CKD include age, gender, inflammation, malnutrition, inactivity, sleep disturbances, cognitive decline, advanced CKD, gut dysregulation, and medications. While we adjusted for major covariates, we cannot exclude the influence of unknown confounding factors, such as sleep quality, stress, biological mechanisms, effect modifiers (hormone levels, inflammation markers, etc.),biochemical data(e.g., vitamin D, PTH, nutrition), medication use, and others, which limit the study. Finally, the data from this study, spanning from 2005 to 2018, may lead to an underestimation of the current prevalence and treatment methods for sleep-related issues, thus potentially limiting the strength of the findings.

## 5. Conclusion

This study explored a nationally representative dataset to identify a close association between sleep health and frailty in CKD patients. Our innovative findings suggested an association between short or long sleep duration, trouble sleeping, and frailty in CKD patients emphasizing the importance of considering multiple dimensions of sleep health when addressing health outcomes including frailty symptoms in CKD patients.

## Supplementary Material

Supplementary Table 1 Detailed scoring criteria for 49 vulnerability indices.docx

Fig3_Forest_plot_of_interaction_effect_of_sleep_duration_and_trouble_sleeping_for_frailty.jpeg

Supplementary Material（ Covariates and their evaluation）.docx

Supplementary Table3.docx

Fig2_log_odds_between_sleep_duration_and_Frailty.jpeg

Supplementary Table 2.docx

Fig1_Flow_chart_of_sample_selection.jpeg

## Data Availability

All data generated or analyzed in this study are available from the National Health and Nutrition Examination Survey (NHANES). https://www.cdc.gov/nchs/nhanes/index.htm.
