# Supplementary figures and images for: Interactive effect of sleep duration and trouble sleeping on frailty in chronic kidney disease: findings from NHANES, 2005–2018
Source: Ren Fail. 2025 Feb 27;47(1):2471008. doi: 10.1080/0886022X.2025.2471008 (PMC11869335; doi:10.1080/0886022X.2025.2471008)

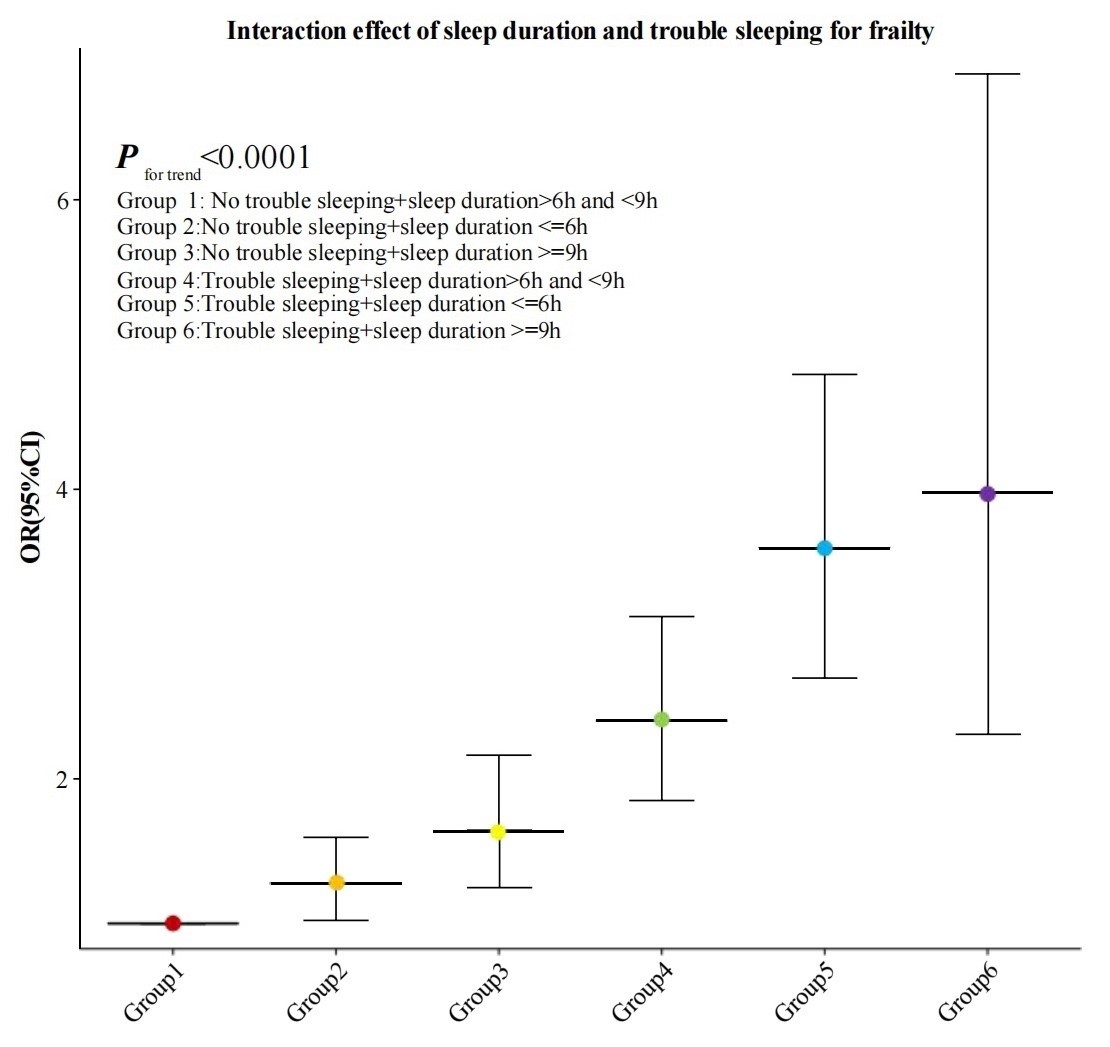

Supplement: Fig3_Forest_plot_of_interaction_effect_of_sleep_duration_and_trouble_sleeping_for_frailty.jpeg [file IRNF_A_2471008_SM6059.jpeg]

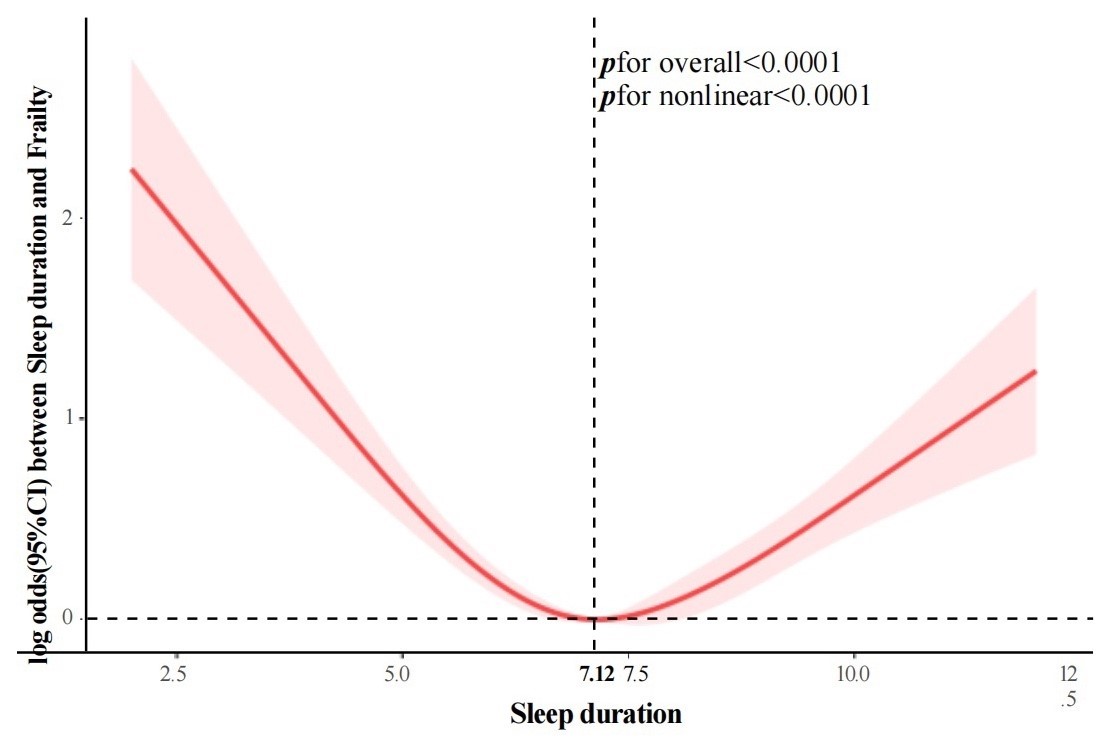

Supplement: Fig2_log_odds_between_sleep_duration_and_Frailty.jpeg [file IRNF_A_2471008_SM6056.jpeg]

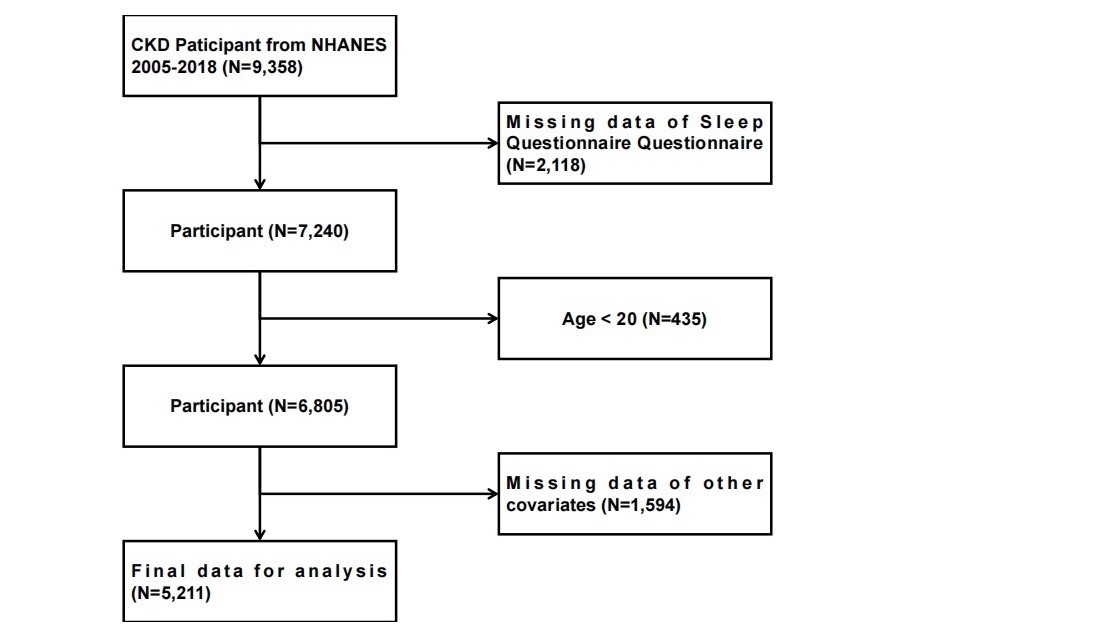

Supplement: Fig1_Flow_chart_of_sample_selection.jpeg [file IRNF_A_2471008_SM6054.jpeg]
