# Supplementary material for: Interactive effect of sleep duration and trouble sleeping on frailty in chronic kidney disease: findings from NHANES, 2005–2018
Source: Ren Fail. 2025 Feb 27;47(1):2471008. doi: 10.1080/0886022X.2025.2471008 (PMC11869335; doi:10.1080/0886022X.2025.2471008)
Supplement: Supplementary Material（ Covariates and their evaluation）.docx [file IRNF_A_2471008_SM6058.docx]

Covariates and their evaluation

（1）Race include Mexican Americans, non-Hispanic blacks, non-Hispanic whites, other Hispanics, and other races.

（2）Poverty Income Ratio（PIR）:The PIR was derived by dividing household income by the poverty threshold.

（3）Body mass index（BMI）：BMI was obtained from dividing weight in kilograms (kg) by the square of height in meters(m2).

（4）Marital Status was categorized as married or living with a partner or not.

（5）Education levels were coded as less than high school and graduated high school or more.

（6）Smoking status were grouped into three categories: never (smoked less than 100 cigarettes in life),former (smoked more than 100 cigarettes in life and smoke not at all now), now (smoked more than 100 cigarettes in life and smoke some days or every day).

（7）Drinking status was classified as either never drinking or drinking.

（8）Physical activity is classified as vigorous physical activity and moderate physical activity.Vigorous physical activity was defined as,“large increases in breathing or heart rate and is done for at least 10 min continuously”，and moderate physical activity was defined as, “small increases in breathing or heart rate and is done for at least 10 min continuously”.Participants reported their activity over the previous 30 days in the 2005-2006 cycles and over the course of a week in the 2007-2014 cycle.

（9）Diabetes can be diagnosed if one of the following 6 criteria is conformed：

1.doctor told you have diabetes；

2.glycohemoglobin HbA1c(%) >= 6.5；

3.fasting glucose(mmol/l) >= 7.0；

4.random blood glucose (mmol/l) >= 11.1；

5.two-hour OGTT blood glucose (mmol/l) >= 11.1；

6.Use of diabetes medication or insulin.

（10）Diagnosis of hypertension：

1.doctor told you have hypertension；

2.average blood pressure abnormal；

3.Use anti-hypertension drugs.

The average blood pressure is calculated as：

•The diastolic reading with zero is not used to calculate the diastolic average.

•If all diastolic readings were zero, then the average would be zero.

•If only one blood pressure reading was obtained, that reading is the average.

•If there is more than one blood pressure reading, the first reading is always excluded from the average.
