# Supplementary material for: Interactive effect of sleep duration and trouble sleeping on frailty in chronic kidney disease: findings from NHANES, 2005–2018
Source: Ren Fail. 2025 Feb 27;47(1):2471008. doi: 10.1080/0886022X.2025.2471008 (PMC11869335; doi:10.1080/0886022X.2025.2471008)
Supplement: Supplementary Table 2.docx [file IRNF_A_2471008_SM6055.docx]

|  | **Model 5^e^**  **OR (95%CI) P-value** | **Model 6^f^**  **OR (95%CI) P-value** | **Model 7^g^**  **OR (95%CI) P-value** | **Model 8^h^**  **OR (95%CI) P-value** | **Model 9^i^**  **OR (95%CI) P-value** |
| --- | --- | --- | --- | --- | --- |
| **Sleep duration** | 0.957 (0.907, 1.010) 0.1155 | 0.966 (0.917, 1.017) 0.1859 | 0.967 (0.918, 1.018) 0.2053 | 0.958 (0.907, 1.011) 0.1182 | 0.931 (0.872, 0.995) 0.0413 |
| **Sleep duration** |  |  |  |  |  |
| **>6, and <9** | Ref. | Ref. | Ref. | Ref. | Ref. |
| **<=6** | 1.527 (1.274, 1.832) <0.0001 | 1.508 (1.264, 1.800) <0.0001 | 1.488 (1.255, 1.765) <0.0001 | 1.497 (1.253, 1.789) <0.0001 | 1.630 (1.330, 1.997) <0.0001 |
| **>=9** | 1.571 (1.198, 2.060) 0.0015 | 1.587 (1.228, 2.052) 0.0007 | 1.588 (1.234, 2.044) 0.0006 | 1.557 (1.196, 2.026) 0.0015 | 1.615 (1.155, 2.257) 0.0073 |
| **Trouble**  **sleeping** |  |  |  |  |  |
| **No** | Ref. | Ref. | Ref. | Ref. | Ref. |
| **Yes** | 2.539 (2.073, 3.110) <0.0001 | 2.543 (2.070, 3.124) <0.0001 | 2.508 (2.046, 3.075) <0.0001 | 2.528 (2.062, 3.099) <0.0001 | 2.390 (1.833, 3.117) <0.0001 |

1. Unadjusted model.
2. Adjusted for age, sex, race, marital status, PIR, and educational level.
3. Adjusted for covariates in Model 2 and smoke, drinking, vigorous physical activity, moderate physical activity, BMI, diabetes, hypertension
4. In addition to all covariates included in model 3, sleep duration and trouble sleeping were each adjusted for the other
5. In addition to all covariates included in model 4 and CKD stage
6. In addition to all covariates included in model 5 and serum phosphorus, serum calcium, serum potassium
7. In addition to all covariates included in model 6 and TG, TC, HDL
8. In addition to all covariates included in model 7 and Osteoporosis, Hyperlipidemia
9. Exclusion of dialysis patient
